# Molecular Analysis of the *Heterakis dispar* Population in Domestic Geese Based on the ITS1-5.8rRNA-ITS2 Fragment

**DOI:** 10.3390/ani12070926

**Published:** 2022-04-04

**Authors:** Kamila Bobrek, Andrzej Gaweł, Joanna Urbanowicz

**Affiliations:** Department of Epizootiology and Clinic of Bird and Exotic Animals, Faculty of Veterinary Medicine, Wrocław University of Environmental and Life Sciences, Grunwaldzki 45, 50-366 Wrocław, Poland; andrzej.gawel@upwr.edu.pl (A.G.); joanna.urbanowicz11@gmail.com (J.U.)

**Keywords:** *Heterakis dispar*, nematode, ITS1-5.8rRNA-ITS2, geese

## Abstract

**Simple Summary:**

Heterakis is a nematode which infects the ceca of birds. In geese, *Heterakis dispar* is the most common species. Due to the scarcity of data concerning the *H. dispar* population, we are providing this analysis based on the ITS1-5.8rRNA-ITS2 region, one of the most frequently used fragments in genetic analysis. We analyzed 71 *H. dispar* specimens isolated from 20 geese flocks. In the analyzed fragment, four nucleotide differences were noted, resulting in six types of sequences (A, B, C, D, E, and F). The most frequently noted type was type A (45%), followed by type B (18.3%), type C and D (11.3%), type E (8.5%), and finally, type F (5.6%). Infection with nematodes from different types of groups was noted in 30% of flocks, with type A being the most prevalent, followed by types B, D, or E to make up the remaining 100%. This study represents the first *H. dispar* population analysis based on the ITS1-5.8rRNA-ITS2 fragment.

**Abstract:**

Heterakidosis is a parasitic infection in birds caused by the cecal parasite *Heterakis* spp. The most common species in geese is *H. dispar*, the largest avian heterakids species. Because of a scarcity of data concerning the *H. dispar* population, the aim of this study was the genetic analysis of *Heterakis dispar* isolated from geese flocks based on the ITS1-5.8rRNA-ITS2 fragment. Among the 71 *H. dispar* specimens isolated from 20 geese flocks, six haplotypes were determined (A, B, C, D, E, and F). The four nucleotide substitutions were noted in both ITS fragments, and all of them were transitions between adenine and guanine, or thymine and cytosine. The most frequently noted haplotype was type A (45%), followed by type B (18.3%), type C and D (11.3%), type E (8.5%), and F (5.6%). Infection with nematodes from different haplotype groups was noted in 30% of the flocks, with type A being the most prevalent, followed by types B, D, or E to make up 100%. This study represents the first *H. dispar* population analysis based on the ITS1-5.8rRNA-ITS2 fragment.

## 1. Introduction

Heterakidosis is a parasitic infection in birds caused by the cecal parasite *Heterakis* species (Nematoda, Secernentea, Ascaridida). In geese, the most often noticed hetarakid is *Heterakis dispar* [1,2], the largest avian heterakid species. Our previous research has shown that almost 36% of parental geese flocks were infected with *Heterakis dispar*, which results in a high percentage of infestation [1]. The ITS1-5.8rRNA-ITS2 sequence of *Heterakis dispar*, obtained during our previous research, was the first sequence of this species added to GenBank and was almost identical to that of *H. isolonche*, and demonstrated a close similarity to other heterakids (87–88%) [3]. The lack of other ITS1-5.8rRNA-ITS2 sequences of *Heterakis dispar*, and the complete lack of data on the population structure have influenced the next step of the investigation, whose aim was the genetic analysis of *Heterakis dispar* isolated from parental geese flocks.

## 2. Materials and Methods

### 2.1. Parasites Collection

Adult nematodes were collected from the ceca of geese from 20 reproductive flocks which were delivered to the Department of Epizootiology and Clinic of Bird and Exotic Animals at the Faculty of Veterinary Medicine in Wrocław. All birds were delivered following their natural death, for routine necropsy and diagnostic analysis [1]. During the necropsy, the intestinal tract of each bird was opened and the cecal content was collected into Falcon tubes in order to inspect it for the presence of parasites. The nematodes were isolated using a sieve, washed in physiological saline, and identified using the morphological characteristics of males as described previously [3]. All parasites isolated from each goose were counted and subsequently divided after microscopic examination into groups of males and females. All of the isolated males from the geese of one flock were then put together to create a single sample from the aforementioned flock. From this sample, now containing just *Heterakis* males, five were randomly chosen for tests. For DNA extraction, five randomly selected nematode males from each sample were chosen.

### 2.2. DNA Extraction and PCR Reactions

DNA was isolated using a Sherlock AX commercial kit for small amounts of genetic material (A&A Biotechnology, Gdynia, Poland) in accordance with the manufacturer’s instructions. The template genetic material was deposited at −20 °C until use.

The PCR reactions for the ITS-5.8rRNA-ITS2 region were performed using 12.5 μL 2xPCR Master Mix Plus (A&A Biotechnology, Poland); 0.2 μL of both Primer2 Forward and Primer2 Reverse [4]; 2 μL DNA; and RNAse free water (A&A Biotechnology, Poland) for a total volume of 25 μL. The primers used in the PCR reactions were: forward (5′-GTTTCCGTAGGTGAACCTGC-3′) and reverse (5′-ATATGCTTAAGTTCAGCGGGT-3′).

The PCR cycling parameters were as follows: initial denaturation at 95 °C for 10 min, followed by 35 cycles of denaturation at 95 °C for 30 s, primer annealing at 67.6 °C for 1 min, and extension at 72 °C for 1 min. The final extension was at 72 °C for 10 min [4]. 

The PCR products were analyzed via 2% agarose gel staining with Midori Green advance DNA (NIPPON Genetics Europe GmbH, Dueren, Germany) under UV light. The obtained products of about 920 bp in length were isolated from the agarose gel using a Gel Out Concentrator Kit (A&A Biotechnology, Gdynia, Poland) and were subsequently sent to Macrogen (Amsterdam, The Netherlands) for sequencing, with a forward and reverse starter each.

### 2.3. Sequencing and Phylogenetic Analysis

The sequences obtained in this study were edited, and after visual inspection of the chromatograms, the sequences were assembled for each specimen. The received unique sequences have been deposited in the EMBL database under accession numbers OM530142, OM530143, OM530144, OM530145, and OM530147. The sequences were aligned with sequence MF319969 which had been previously deposited (as the only available sequence of the *H. dispar* ITS1-5.8rRNA-ITS2 fragment) in the National Center for Biotechnology Information (NCBI) GenBank database using CLUSTAL W in the MEGA7 package. Phylogenetic analyses were performed using MEGA7 software. The phylogenetic tree was inferred by using the maximum likelihood method.

## 3. Results 

Among one hundred *H. dispar* males intended for genetic analysis, two correct sequences from the 3′ end and 5′ end were obtained from 71 males. The ITS1-5.8S-ITS2 PCR products were about 920 bp; after cutting the dubious ends of the sequences and assembling each specimen, sequences of 888 nucleotides in length—the ITS1-5.8S-ITS2 fragments—were obtained. Among the *H. dispar* specimens, six types of sequences were determined and given the names of A, B, C, D, E, and F corresponding to the six haplotypes. The previously deposited GenBank database sequence (MF319969) named type B has been defined as a reference for the other *H. dispar* sequences obtained in this study [3]. The molecular analysis showed four substitutions, of which all were transitions between adenine and guanine, or thymine and cytosine (Table 1). The substitutions were denoted in the ITS1 regions as nucleotides 47 and 176, and nucleotides 728 and 780 in the ITS2 fragment. 

The most frequently noted ITS1-5.8S-ITS2 haplotype was type A (32/71), which was found in 45% of male nematodes followed by type B (18.3%), type C and D (11.3%), type E (8.5%), and F (5.6%). Among the 20 flocks, invasion with nematodes of different haplotypes was noted in six flocks, with type A dominating among them. In three of these, combinations of types A and B were detected, in two flocks, combinations of types A and D were noted, and one flock was infected with *H. dispar* types A and E.

**Figure 1:**
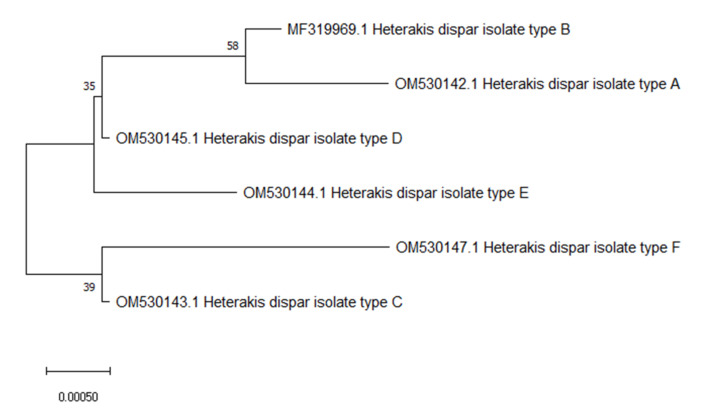
Phylogenetic tree interrelationships among *Heterakis dispar* haplotypes based on maximum likelihood method with 1000 bootstrap randomizations of partial ITS1-5.8S2-ITS2 sequence (888 bp). The scale bar indicates the number of substitutions per site.

In an attempt to create a phylogenetic tree of the *H. dispar* haplotypes, we performed a molecular analysis using the maximum likelihood method based on the ITS1-5.8S2-ITS2 fragment. The relationship between the *H. dispar* haplotypes is visualized in Figure 1. The tree revealed two clearly separate clades in *Heterakis dispar* sequences: one included haplotypes A, B, C, and D, while the second contained haplotypes E and F (Figure 1). The haplotypes A and B vary by just one nucleotide, which makes this substitution the most probable, and the most common. Between haplotype A and the other haplotypes, 2 or 3 single nucleotide variations were observed (Table 1).

**Figure 2:**
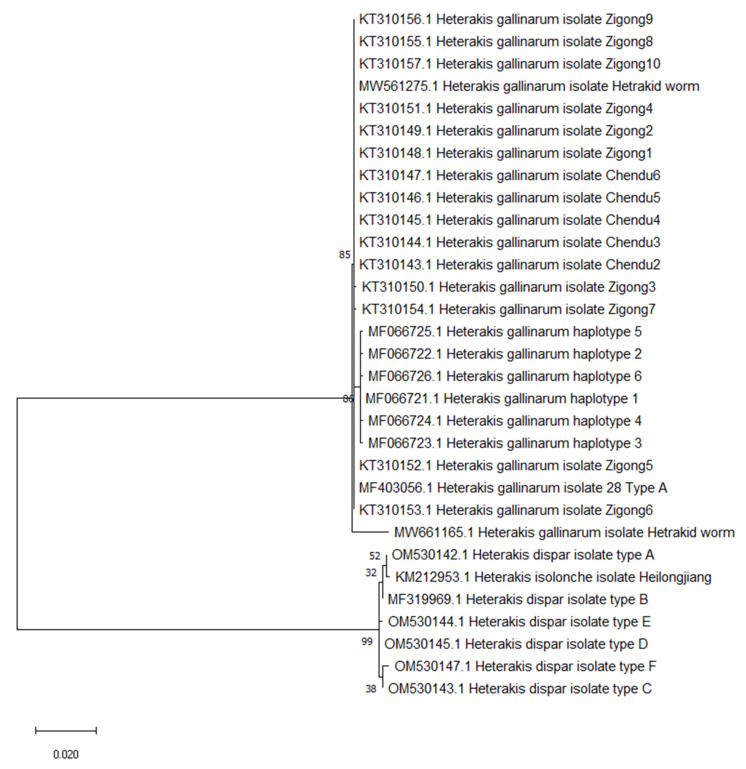
Phylogenetic tree interrelationships among poultry *Heterakis* spp. on maximum likelihood method with 1000 bootstrap randomizations of partial ITS1-5.8S2-ITS2 sequence (888 bp). The scale bar indicates the number of substitutions per site.

The relationship between all available GenBank sequences of poultry *Heterakis* spp. is shown in Figure 2. The *Heterakis gallinarum* sequences form a clearly separated clade, and a second clade is formed by *H. dispar* and *H. isolonche* sequences.

## 4. Discussion

Heterakidosis and its cause in gallinaceous birds, *H. gallinarum*, is well described in terms of its morphological and molecular characteristics. Information about *Heterakis* infection in waterfowl is scarce and no molecular studies based on the ITS1-5.8S-ITS2 region are available. The ITS fragments, highly conserved flanking sequences with a high copy number in genetic material, are one of the most frequently used fragments for genetic analysis due to their interspecific levels of variability [5]. In this research, six different haplotypes of *Heterakis dispar* were defined, and the ITS1-5.8S-ITS2 fragments obtained by our team were the first official sequences of this part of the genome placed in GenBank. The haplotypes varied in four nucleotide positions and, in those variants, transversion occurred. Similar results were achieved by Tunisian scientists [6], who analyzed *Heterakis gallinarum* ITS1-5.8S-ITS2 sequences. They also defined six haplotypes, with five nucleotide substitutions—three transversions and two transitions among the analyzed *H. gallinarum* isolates. Comparing those sequences with a Polish isolate (MF403056) used as the outgroup for *H. dispar* phylogenetic analysis, two other unique transversions were noted in *H. gallinarum*. This suggests that the diversity of haplotypes in *Heterakis* ITS1-5.8S-ITS2 fragments can vary between countries. Amor et al. [6] emphasizes the presence of differences between the haplotypes due to wider geographic distances. Despite the local variability, within the *Heterakis gallinarum* population, ITS identity is high. The low genetic diversity among *Heterakis* isolates from chickens was also confirmed in Egypt [4]. These observations concerning the low variability in Heterakids ITS fragments are consistent with data among other nematode species such as *Haemonchus contortus, Baylisascaris schroederi*, *Bursaphelenchus* spp., and *Hoplolaimus* spp. [7,8,9,10].

In our study, infections with parasites from different haplogroups were observed in 30% of flocks. The most common combination, present in 50% of flocks infected with varied haplotypes, was when haplotypes varied by just one nucleotide. The single nucleotide substitution in one position of the analyzed fragment is the most probable mutation which can occur and is one of the most useful and widely applied markers in genetic studies [11].

The occurrence of different haplotypes of parasites in a group of animals or even in one host is not rare in animals. The various ITS haplotypes of *H. contortus* were noted in blue sheep in China [9], and in Mongolian goats [12]. In commercial geese production, animals still need access to pastures, which facilitates parasitic infections. In theory, gene flow in parasite populations is determined by the parasites’ own history and the hosts’ movements. Parental flocks of geese live a few years, and despite regular deworming, contact with the environment favors nematode egg intake, which may be shed by the carriers—domestic or wild geese.

The phylogenetic analysis showed that *H. gallinarum* sequences form clearly separated clades and there are no obvious geographical varieties among them. The Polish isolate MF403056 was homologous to Chinese isolates, and varied from Tunisian isolates by just two or three nucleotides. A second clade was formed by *Heterakis dispar* and *Heterakis isolonche,* homologous to the *H. dispar* haplotype A. The *H. isolonche* specimen was isolated from the Rhine goose, which may suggest that parasites of the same host could present similar genetic sequences, or that this specimen was not *H. isolonche*, but *H.dispar.* To discuss the homology of *H. isolonche* and *H. dispar*, future investigations and more sequences are needed.

## 5. Conclusions

This research showed the presence of six haplotypes in a *Heterakis dispar* population based on the ITS1-5.8S-ITS2 fragment. The most frequently noted haplotype was type A (45%), followed by type B (18.3%), type C and D (11.3%), type E (8.5%), and F (5.6%). In 70% of the flocks, the infection was caused by one haplotype, and in the rest of the flocks, two haplotypes were confirmed, with type A being the most dominant, followed by types B, D, or E to make up 100%. Our research is the first attempt at the phylogenetic analysis of the *H. dispar* population based on the ITS1-5.8S-ITS2 fragments, and due to a lack of sequences for other isolates, remains the only one.

## Figures and Tables

**Table 1:** Types of *Heterakis dispar* based on the differences in the ITS1-5.8rRNA-ITS2 fragment.

*Heterakis dispar* Type	GenBank Accession Number	Nucleotide Position in the Obtained Sequences				Number of Parasites (n = 71)
		Pos 47	Pos 176	Pos 728	Pos 780	
B	MF319969	A	C	G	C	13
A	OM530142	G				32
C	OM530143			A	T	8
D	OM530144				T	8
E	OM530145		T		T	6
F	OM530147	G	T	A	T	4

## Data Availability

Not applicable.
